# Morphoanatomical, Physiological, and Biochemical Indicators in *Lactuca sativa* L. Germination and Growth in Response to Fluoride

**DOI:** 10.3390/plants11233406

**Published:** 2022-12-06

**Authors:** Arthur Almeida Rodrigues, Douglas Almeida Rodrigues, Juliana de Fátima Sales, Sebastião Carvalho Vasconcelos Filho, Alan Carlos Costa, Cássia Lino Rodrigues, Adinan Alves da Silva, Marisa Domingos, Caroline Müller

**Affiliations:** 1 Laboratory of Seeds, Goiano Federal Institute of Education, Science and Technology (IFGoiano), Campus Rio Verde, P.O. Box 66, Rio Verde 75901-970, Brazil; 2 Laboratory of Plant Anatomy, Goiano Federal Institute of Education, Science and Technology (IFGoiano), Campus Rio Verde, P.O. Box 66, Rio Verde 75901-970, Brazil; 3 Laboratory of Ecophysiology and Plant Productivity, Goiano Federal Institute of Education, Science and Technology (IFGoiano), Campus Rio Verde, P.O. Box 66, Rio Verde 75901-970, Brazil; 4 Instituto de Botânica, Núcleo de Pesquisa em Ecologia, Miguel Stéfano Ave. 3687, São Paulo 04045-972, Brazil

**Keywords:** chlorophyll *a* fluorescence, germination speed index, oxidative stress, potassium fluoride

## Abstract

Fluoride is one of the main phytotoxic environmental pollutants, and high concentrations (10–30 mg L^−1^) are commonly detected in surface and groundwater. Little, however, is known about the effects of this pollutant on crops that require irrigation during their development, which, in addition to phytotoxicity, may cause negative human health effects. Thus, the aim of this study was to characterize the effects of potassium fluoride (KF) on the germination of lettuce seeds and identify the physiological and anatomical markers of this pollutant’s action on plants exposed to it during growth. Initially, lettuce seeds were sown in gerboxes and soaked in solutions containing 0 mg L^−1^, 10 mg L^−1^, 20 mg L^−1^, and 30 mg L^−1^ KF. Plants grown in a greenhouse were treated daily with KF irrigation at the same KF concentrations for 40 days. KF exposure reduced the germination rate and germination speed index of lettuce seeds at 20 mg L^−1^ and 30 mg L^−1^, resulting in compromised root development at the highest KF concentration. Lettuce plants displayed a slight photosynthesis reduction and a significant photochemical efficiency decrease after exposures to all KF concentrations. Lower chlorophyll contents and nitrogen balance indices were observed in plants exposed to 30 mg L^−1^ KF. On the other hand, increases in phenolic compounds and malondialdehyde were noted with increasing KF concentrations. Lettuce plants can, therefore, accumulate fluoride in leaves when irrigated with KF-rich water. The investigated physiological and biochemical variables were proven to be adequate fluoride action biomarkers in lettuce plants and may become an important tool in the study of olericulture contaminants.

## 1. Introduction

Environmental air pollutant contamination has increased due to industrialization advancements, among other human activities, leading to severe deleterious effects on both living beings and ecosystems [1]. Among a range of known pollutants, fluoride (F), a by-product generated in the manufacturing practices of aluminum, ceramics, phosphate fertilizers, and bricks [2], is noteworthy. At high concentrations, this element causes serious human health problems, such as dental and bone fluorosis [3], and it is also one of the most toxic plant pollutants [4,5].

Despite being released mainly into the atmosphere, water is an important means of environmental F contamination. In many areas worldwide, water sources containing high F concentrations are a serious public health threat [6,7]. This contaminant can be incorporated into the water through excess atmospheric releases, which return this element to the surface in the form of rain containing high F levels, as well as through contaminated effluent dumping in water bodies and agricultural activities [8].

According to the World Health Organization, the maximum permitted F limit in drinking water is 1.5 mg L^−1^ [9], a limit also established by the Environmental Agency of the State of São Paulo [10] in Brazil. However, aquifers and water bodies often contain F concentrations that exceed this limit. Fluoride concentrations worldwide have been reported to be higher than 1.5 mg L^−1^, reaching up to 40 mg L^−1^ [11,12,13]. In Brazil, CETESB identified F contaminations above the recommended limit in water wells in the state of São Paulo, reaching up to 10 mg L^−1^ [14].

When released into the environment, F can come into contact with plants in several ways. The main gaseous F plant absorption route is through leaf stomata [15], while aqueous F can be incorporated through entire leaf surfaces [16,17]. Upon entering the plant, F moves through apoplastic routes, reaching leaf margins and the apex. It may also be accumulated in the mesophyll, causing the collapse of parenchymal tissues and subsequent visual symptoms, such as chlorosis and necrosis [18,19]. The physiological and metabolic effects of this element include photosynthetic metabolism alterations [20,21], reactive oxygen species (ROS) formation, the triggering of oxidative stress [22], and mineral metabolism damage, mainly due to the ability of this pollutant to form Ca^2+^, Mg^2+^, and Mg^2+^ complexes, leading to nutritional deficiency [23].

The presence of F in the environment, mainly in surface and underground waters used for irrigation, can directly affect seed germination and subsequent plant development [24,25,26]. This is due to the impairment of carbohydrate mobilization for adequate growth and the development of the embryonic axis [27], as well as root protrusion inhibition [1]. On the other hand, F effects are reduced in accumulating plants, and this element may, therefore, accumulate in vegetative tissues and edible cereal grains, directly threatening the food chain [28].

The practice of agricultural irrigation is vital for food production worldwide and comprises an important tool to ensure food security for human populations [29]. However, the use of contaminated surfaces and groundwater exposes plants to the risk of pollutant absorption, threatening food security. This can occur with several vegetables, such as lettuce (*Lactuca sativa* L.) [30,31,32], a leafy vegetable widely used in human nutrition as a source of fibers and vitamins, and a very useful species in ecotoxicology bioassays as it provides reliable, fast, and low-cost results which do not require expensive equipment. It is also fast-growing and highly sensitive, often being used as a model species in germination studies [33,34]. However, little information is available concerning F accumulation and its effects on lettuce, which is commonly grown under irrigation conditions using water from water bodies and artesian wells containing unknown F contamination levels.

In this context, lettuce plants were used to characterize the toxic effects of F absorption on seed germination and on the physiology and anatomy of leafy vegetables. This will help to demonstrate the importance of understanding pollutant contents in water used for plant irrigation in order to avoid the consumption of food items containing toxic F levels. Therefore, the aim of this study was to identify the anatomical, physiological, and biochemical indicators related to water-solubilized potassium fluoride action on *Lactuca sativa* germination and growth.

## 2. Results

### 2.1. Germination and Initial Growth of L. sativa Seedlings

Both the total germination (TG) and the germination rate index (GRI) of the *L. sativa* were reduced by 7.5% after exposure to 20 KF mg L^−1^, and by 9.1% after exposure to 30 mg KF L^−1^, compared with the control group (Table 1). Root length was 21.8% shorter in the seedlings exposed to the highest KF concentration, compared with the control group (Table 1).

### 2.2. Greenhouse Experiment

#### 2.2.1. Visible Symptoms

Visual assessments of leaves after KF exposure indicated no visible toxicity symptoms, regardless of the KF concentration. Leaf images 40 days after the beginning of the KF treatments are presented as Supplementary Materials (Figure S1, Supplementary Materials).

#### 2.2.2. Fluoride Content

**Figure 1:**
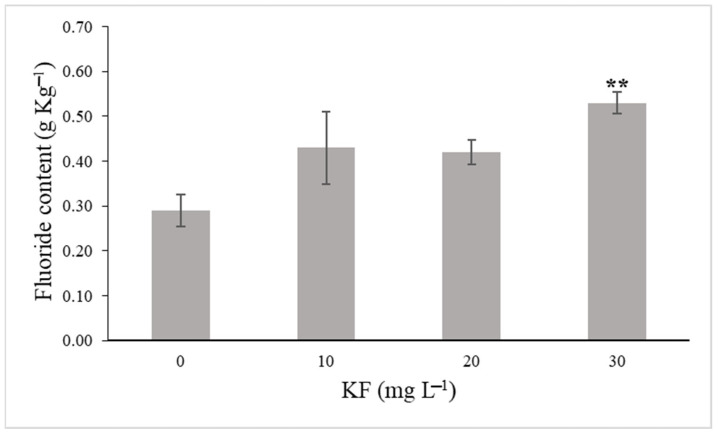
Fluoride (F) content in *Lactuca sativa* L. leaves after 40 days of exposure to 0 mg L^−1^, 10 mg L^−1^, 20 mg L^−1^, and 30 mg L^−1^ potassium fluoride (KF). Data represent the means ± SE (n = 4). Asterisks indicate significant differences at 1% (**) probabilities between the KF treatments and the control group according to the Dunnett test.

The fluoride content (F) of the *L. sativa* leaves increased significantly by 82.8% after exposure to 30 mg KF L^−1^ for 40 days (Figure 1). Even with a slight increase, the other KF treatments showed no significant difference compared with the control (Figure 1).

#### 2.2.3. Physiological Traits

The potential PSII quantum yield (F_v_/F_m_) of *L. sativa* plants was not changed by any of the KF concentrations (Table 2). Significant decreases of up 42.1% and 43.2% were observed in the effective PSII quantum yield (Y_II_) and the electron transport rate (ETR), respectively, in plants exposed to 20 mg KF L^−1^, compared with the control (Table 2). The photochemical extinction coefficient (qL) decreased by 36.8% after exposure to the highest KF doses (20 mg KF L^−1^ and 30 mg KF L^−1^). The regulated non-photochemical quenching (YNPQ) of the *L. sativa* plants was not affected by the KF.

Although not significant, the net photosynthetic rate (*A*) and the carboxylation efficiency (*A*/Ci) reduced by 22.1% and 22.3%, respectively, in plants exposed to 20 mg KF L^−1^. Stomatal conductance (*g*_S_), transpiration (*E*), and the relationship between internal and external CO_2_ concentration (*Ci/Ca*) showed no significant differences after exposure to KF solutions (Table 2).

#### 2.2.4. Biochemical Traits

Chlorophyll (Chl) content was reduced by 26.1% and 21.1% in *L. sativa* plants exposed to 20 mg KF L^−1^ and 30 mg KF L^−1^, respectively (Table 3). Flavonol (Flv) and anthocyanin (Anth) contents were not changed by any of the KF exposures (Table 3). Malondialdehyde (MDA) content increased significantly and gradually with the increasing KF doses, from 44.7% (10 mg L^−1^) to 55.3% (30 mg L^−1^). Hydrogen peroxide (H_2_O_2_) content increased (27.6%) in *L. sativa* plants only at the highest KF concentration (30 mg L^−1^) (Table 3).

#### 2.2.5. Phenolic Compounds in *L. sativa* Roots and Leaves

The *L. sativa* plants showed no phenolic compound accumulation in leaf and root cells in the absence of KF (control) (Figure S2B). On the other hand, a gradual increase in phenolics was observed with increasing KF concentrations (Figure S2) in the *L. sativa* plants treated with the KF solution. Phenolic compounds were verified by black staining in the epidermis and parenchyma cells (Figure S2C–H) (Figure S2, Supplementary Materials).

#### 2.2.6. Leaf Nutrients

The nitrogen balance index (NBI) was reduced by 16.9% in the *L. sativa* leaves exposed to the highest dose of KF (30 mg L^−1^), compared with the control (Table 4). Calcium (Ca), magnesium (Mg), and potassium (K) levels did not change in the *L. sativa* leaves, regardless of the KF dose (Table 4).

#### 2.2.7. Anatomical Leaf and Root Characterizations

**Figure 2:**
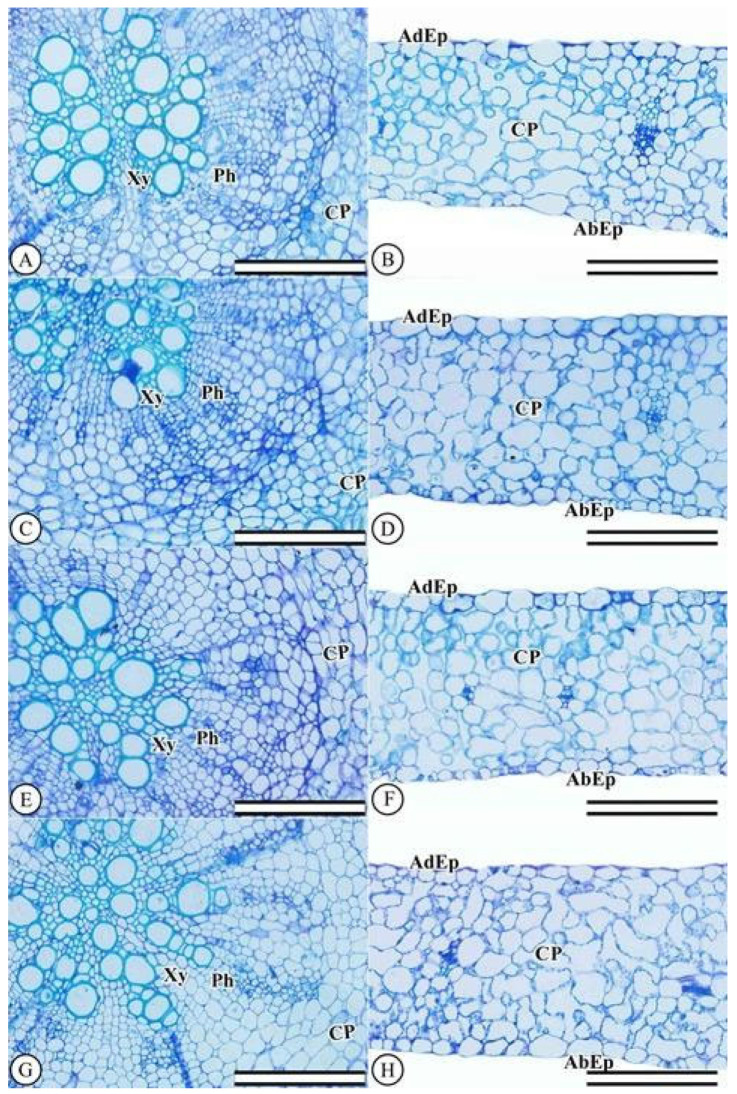
Root and leaf anatomy of *Lactuca sativa* L. plants following 40 days of exposure to potassium fluoride (KF) at different concentrations. (**A**,**B**) control (0 mg L^−1^), (**C**,**D**) 10 mg L^−1^ KF, (**E**,**F**) 20 mg L^−1^ KF, and (**G,H**) 30 mg L^−1^ KF. (EpAd) adaxial epidermis; (EpAb) abaxial epidermis; (CP) chlorophyll parenchyma; (Xy) xylem; (Ph) phloem. scale bar = 200 µm.

The epidermis of *L. sativa* plants is unstratified on both surfaces, with larger epidermal cells on the abaxial surface than on the adaxial face. The mesophyll consists of chlorophyll parenchyma located between the two epidermal surfaces, homogeneously distributed and formed by round cells (Figure 2B,D,F,H). The *L. sativa* leaves displayed no damage in the chlorophyll parenchyma region or in the epidermal cells. The cells were regularly shaped and showed no changes after increasing KF doses. Anatomical characterizations of the roots indicated no cellular changes after increasing KF doses (Figure 2A,C,E,G).

#### 2.2.8. Principal Component Analysis

**Figure 3:**
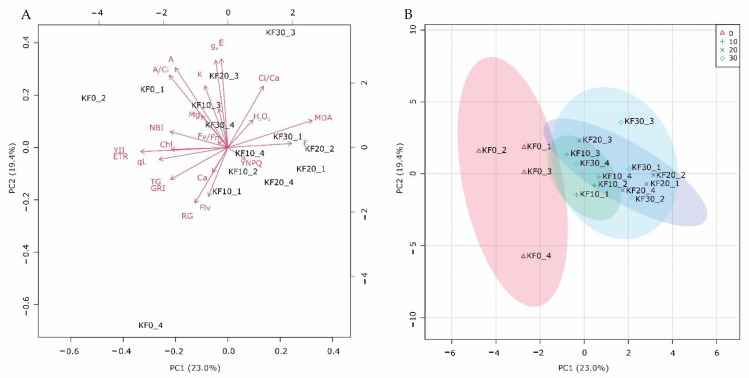
Principal component analysis (**A**) and score plot (**B**) for fluoride, nutrient content, physiological, biochemical, and morphological traits of *Lactuca sativa* L. plants after 7 days of exposure to 0 mg L^−1^, 10 mg L^−1^, 20 mg L^−1^, and 30 mg L^−1^ potassium fluoride (KF) in the germination test and 40 days of cultivation in a greenhouse with 0 mg L^−1^, 10 mg L^−1^, 20 mg L^−1^, and 30 mg L^−1^ KF treatments.

The first three main components explained 54.1% of the total data variation. The greatest contribution to the first component (PC1) comprised fluoride and malonaldehyde, photochemical traits (ETR, YII, *A*, *A*/Ci), NBI, and morphological characteristics, while water characteristics (g_s_, *E*), flavonol, anthocyanin, and root growth contributed more to PC2 (Figure 3A).

The score plot indicated a clear separation between the KF treatments and the controls as well as a high degree of overlap between the different KF concentration treatments (Figure 3B).

## 3. Discussion

Despite significant effects on the germination and initial growth of the seedlings and reductions in the electron transport rate, photosynthesis, and chlorophyll content, as well as an increase in MDA, especially at the highest dose of KF, the *L. sativa* plants did not show changes in their plant anatomy. Inhibitory F effects on seed germination have already been reported for different species, including rice (*Oryza sativa* L.) [1], chickpeas (*Cicer arietinum* L.), and barley (*Hordeum vulgare* L.) [35].

Toxic compounds such as F may interfere with enzymatic plant activity and energy release, which may have been the case here, leading to decreases in total germination percentage, germination speed index, and root length of the *L. sativa* seedlings. Although the mechanisms directly responsible for these responses are not entirely clear, F toxicity towards the cellular metabolism can affect essential germination and the initial growth processes of seedlings, such as ATP production, respiration enzyme activity, nutrient availability, cell expansion, and water and nutrient absorption [36,37,38]. Fluoride seed damage becomes even more relevant when considering that embryo defense mechanisms against cellular damage are still not very efficient. For example, oxidative damage affects membrane permeability and protein structure, leaving the embryo susceptible to structural irregularities, such as cotyledons [39].

The most pronounced KF effects, including root growth, occurred after exposure to higher KF concentrations and greater fluoride absorption. According to Pereira et al. [40], *L. sativa* pericarps and seed integuments can act as a protective barrier against pollutant absorption when available at low medium concentrations. Thus, germination was not affected following 10 mg L^−1^ and 20 mg L^−1^ KF exposures.

*L. sativa* leaves from plants grown under daily exposure to different KF concentrations showed no visible symptoms after 40 days, although pollutant accumulation and physiological changes were detected. In this context, several photochemical changes were observed. KF exposure led to decreased effective PSII quantum yields (YII) and electron transport rates (ETR), regardless of concentration. Decreased chlorophyll content resulting from KF toxicity may have contributed to photochemistry inhibition.

Decreased photochemical efficiency accompanied by a slight reduction in the photochemical extinction coefficient has been reported as being accompanied by decreased photosynthetic pigment concentrations [41], as noted in the present study. In this study, a decrease of up to 17% in the total chlorophyll index was observed following KF exposure. Cai et al. [42] reported a 15% reduction in chlorophyll content after cultivation in fluoride solutions when studying the germination of *Camellia sinensis*. Decreased chlorophyll content can result from pollutant entry into chloroplasts in the form of F ions, which can bind to the central complex of Mg_2_^+^ in the porphyrin ring, thus altering molecule structures.

Fluoride is also known to impair the biosynthesis of carotenoids [43,44], pigments that act in the dissipation of excess energy in the photosynthetic apparatus and prevent photosynthesis photoinhibition. Because of this, there may not have been an increase in energy dissipation in the form of heat, as verified by the absence of Y_NPQ_ variations. However, even with the lack of action of this protection mechanism, the lettuce plants exposed to KF in this study did not present permanent damage to their photochemical apparatus, since the Fv/F_m_ ratio was not significantly altered by the treatments, and they still presented values that indicate the physiological stability of PSII in the face of pollutants [45].

In general, *L. sativa* gas exchanges were not significantly altered by the KF treatments. The slight reduction in the photosynthetic rate (*A*) appears to have resulted from *gs* decreases, indicating a KF effect on the diffusive restriction of photosynthesis, which is even more evident due to the absence of variations in the C_i_/C_a_ and A/C_i_ ratios, which translate potential biochemical damage to the photosynthetic process. Decreased *gs* following F exposure may be a protective mechanism to limit the absorption and accumulation of this pollutant in intercellular spaces. Hoshika et al. [46] demonstrated that stomatal closure may be considered a response to prevent stress caused by atmospheric pollutants, maximizing carbon gain and minimizing water loss and pollutant absorption. These responses may be related to the plant’s ability to reduce the phytotoxic pollutant effects.

Fluoride has been reported to increase plant reactive oxygen species production, causing oxidative stress [47,48,49]. In this study, oxidative damage in the *L. sativa* leaves was noted in the form of increased production of malonaldehyde, a compound that results from membrane lipid peroxidation. Oxidative stress can also compromise electron transport in chloroplasts and reduce the PSII quantum yield by oxidizing proteins and membranes in the photochemical apparatus [50]. The significant reduction in Y_II_ and ETR in this study denotes these types of responses to KF in *L. sativa*.

Plants display a range of antioxidant resources to control oxidative stress [47]. Flavonols are a group of secondary metabolites, which, alongside anthocyanin pigments, play antioxidant roles in plants [51]. Anthocyanins are, in fact, important photoprotective chloroplast compounds [52,53]. However, KF did not induce an increase in these metabolites in the *L. sativa*, suggesting that other antioxidant pathways may have been activated as a primary response to stress. In fact, secondary metabolites can act as a complementary antioxidant mechanism when antioxidant enzyme activities are reduced [54].

Fluoride accumulation in plants can cause a decreased mineral metabolism; in particular, the deficiency or complexation of Ca^+2^ and Mg^2+^ [55]. This deficiency can be explained by F absorption, which in turn reacts with Ca^+2^ to form CaF_2_, which is relatively insoluble, restricting its plant absorption availability [2,56,57]. However, the *L. sativa* leaves did not exhibit altered macronutrient Ca, Mg, or K levels following KF exposure. Concerning NBI decreases in leaves, this may be associated with leaf nitrogen content limitations, as NBI is used as a plant nitrogen nutrition status indicator [58]. The decreased chlorophyll content observed in the *L. sativa* may be the cause of the reductions noted in NBI following the KF exposure, as leaf levels of N are directly related to the levels of these pigments [59].

Morphoanatomical changes in *L. sativa* plants, such as cell hypertrophy and loss of turgor following F exposure can be detected by microscopy techniques, and because they may precede visual symptoms they are considered important prognostic injury indicators [60,61]. However, no cellular changes in the leaf mesophyll were observed in the present study, indicating KF tolerance at the tested concentrations. Phenolic accumulation was detected by the ferrous sulfate histochemical test following exposure to 30 mg L^−1^ KF, suggesting an antioxidant defense mechanism activated by stress-inducing factors [62].

The principal component analysis corroborated the influence of KF on the *L. sativa* responses, separating the KF treatments from the control group. Additionally, this statistical assessment reinforced the proposition that the photosynthetic process variables alongside the NBI, mainly due to PC1, were the most relevant as evidence of KF effects, thus comprising potential physiological indicators of KF toxicity in *L. sativa* plants.

The results reported herein demonstrate that F accumulation in lettuce leaves is a food health problem as this pollutant is readily absorbed from the gastrointestinal tract, with absorption estimates ranging from 75% to 100% [63,64]. The upper limit of F intake for all sources has been established as 0.12 mg kg body weight per day [65] (8.4 mg for a 70 kg person). As the F-exposed plants did not present visual damage that would contribute to the identification of F contamination, laboratory analyses are required to identify damage indicative of the presence of this pollutant, thus increasing the risks of toxic food ingestion. We therefore indicate that further assessments are required to evaluate the effects of this pollutant on food security.

## 4. Material and Methods

Two experiments, one conducted in a growth chamber and the second in a greenhouse, were carried out to evaluate the effects of different potassium fluoride (KF) concentrations on Vanda lettuce (*Lactuca sativa* L.) germination and growth rates. KF solutions were prepared at 0 mg L^−1^ (control), 10 mg L^−1^, 20 mg L^−1^, and 30 mg L^−1^ using distilled water, according to previously reported surface and groundwater concentrations [13,14]. All pH values were adjusted to 6.0 using HCl (2 M) and NaOH (2 M).

### 4.1. Germination Bioassay: Experimental Design and Evaluations

The germination experiment was carried out by applying a completely randomized design consisting of four KF concentrations (0 mg L^−1^, 10 mg L^−1^, 20 mg L^−1^, and 30 mg L^−1^) and four replicates at the Seed Laboratory belonging to the Instituto Federal Goiano (IFGoiano), Campus Rio Verde, Goiás, Brazil. Twenty-five *Lactuca sativa* L. (cv. Vanda, Sakata, Bragança Paulista, Brazil) seeds were placed in a gerbox containing paper sheets moistened with 2.5 times the dry mass (DM) of the seeds and with 0 mg L^−1^ (control), 10 mg L^−1^, 20 mg L^−1^, and 30 mg L^−1^ KF solutions. The gerboxes were maintained in a growth chamber at a constant temperature (25 ± 0.5 °C) and under a 16/8 h (day/night) photoperiod.

Germinations were evaluated daily for 7 days, and, in accordance with BRASIL [66], seeds were considered germinated when a 2 mm root protrusion was observed. At the end of the experimental period, total germination (TG) percentages and germination rate indexes (GRIs) were obtained. The GRIs were obtained by calculating the sum of seeds that germinated each day divided by the number of days, in accordance with Maguire [67], and root length (RL) was measured.

### 4.2. Greenhouse Experiment

#### 4.2.1. Experimental Design and Evaluations

All experiments were conducted in a greenhouse at the IF Goiano campus (17°48′16″ S, 50°54′19″ W, 753 m), under controlled temperature (27 ± 5 °C and 24 ± 5 °C day and night, respectively) and relative humidity (~63 ± 5%) conditions. The *L. sativa* seeds were first germinated in pots containing Bioplant plus substrate (Bioplant, Nova Ponte, Brazil). After their emergence, the seedlings with uniform heights were then transplanted into 5 dm^3^ pots containing the same substrate and fertilized weekly with a nutrient solution containing 1.87g N pot^−1^ (NH_4_NO_3_ and KNO_3_), 0.95 g P pot ^−1^ (KH_2_PO_4_), and 2.31 g K pot^−1^ (KNO_3_ and KH_2_PO_4_) throughout the assay period [68]. All KF applications of 0 mg L^−1^ (control), 10 mg L^−1^, 20 mg L^−1^, and 30 mg L^−1^ began five days after transplanting. The pH values were adjusted to 6.0 using HCl 2M and NaOH 2M. Fluoride applications were carried out by simulating rain with manual sprinklers, applying 50 mL day^−1^ to the aerial portions in accordance with Rodrigues et al. [69]. Substrate irrigation was performed assuming 65% pot capacity.

After 40 days of daily KF exposure, visual and physiological assessments were carried out and the plant material was sampled for further biochemical, morphoanatomical, nutritional, and fluoride analyses.

A completely randomized design comprising four treatments (KF concentrations) and four repetitions was carried out, with each experimental unit consisting of one pot containing two plants (eight plants per treatment).

#### 4.2.2. Fluoride Determinations in Seeds and Plants

Fluoride determinations were carried out in the *L. sativa* leaves from the greenhouse test. The previously dried and ground leaves (2.0 g) were mixed with 8.0 g of sodium carbonate in nickel crucibles and maintained in a muffle furnace at 450 °C for 5 h, and the fluorine was determined in accordance with Rodrigues et al. [69].

#### 4.2.3. Visible Symptoms

Visible KF exposure symptoms were characterized using photographs of the entire leaf area taken with a digital camera (Cyber-Shot HX100V, Sony, Tokyo, Japan) at the end of the experimental period.

#### 4.2.4. Physiological Traits

Chlorophyll *a* fluorescence was determined using an infrared gas analyzer (IRGA; LI-6400xt, Li-Cor, Nebraska, USA) coupled to a leaf chamber fluorometer (6400-40, Li-Cor, Nebraska, USA). The maximum photosystem II quantum yield (PSII) (F_v_/F_m_) was measured after 30 min of dark adaptation. After sample illumination (~1000 µmol m^−2^ s^−1^) for 40 s, a saturation pulse was applied to determine the following light acclimation variables: effective PSII quantum yield (Y_II_), electron transport rate (ETR), photochemical extinction coefficient (q_L_)—which reflects the fraction of open PSII reaction centers [70]—and the quenching of regulated non-photochemical dissipation (Y_NPQ_).

Gas exchanges were evaluated in fully expanded leaves in the same region used to obtain the chlorophyll *a* fluorescence data in order to determine the photosynthetic rate (*A*, µmol m^−2^ s^−1^), stomatal conductance (*g*_s_, mol m^−2^ s^−1^), transpiration (*E*, mmol m^−2^ s^−1^), and the relationship between internal and external CO_2_ concentrations (*C*_i_/*C*_a_). These data were then used to determine instant carboxylation efficiency (*A/Ci*) [71,72]. All evaluations were carried out between 9:00 a.m. and 11:00 a.m. using the IRGA under controlled conditions in the leaf chamber, maintaining a constant active photosynthetic radiation (PAR, 1500 μmol of photons m m^−2^ s^−1^), CO_2_ concentration (Ca, 400 μmol mol^−1^), temperature (25 °C), and relative humidity (50%).

#### 4.2.5. Biochemical Traits

Malonaldehyde (MDA) and hydrogen peroxide (H_2_O_2_) were quantified in fresh material (FM) (leaf samples), previously homogenized in liquid nitrogen on the 40th assay day to evaluate potential cell damage. For the MDA determinations, leaf samples (200 mg) were homogenized in a trichloroacetic solution (TCA; 0.1% *w*/*v*), in accordance with Heath and Packer [73] as adapted by Cakmak and Horst [74]. Aliquots of the previously centrifuged homogenates were added to the reaction solution (4% TBA diluted in 20% TCA) at 95 °C for 30 min. MDA–TBA complexes were determined at 532 nm, discounting the value for non-specific absorbance at 600 nm [73], quantified using an extinction coefficient of 155 mM^−1^ cm^−1^, and expressed as nmol g^−1^ DM.

For the H_2_O_2_ determinations, the leaf samples (200 mg) were homogenized in a potassium phosphate buffer (50 mM, pH 6.5) containing hydroxylamine (1 mM). After filtration and centrifugation, the supernatant aliquots were added to a reaction medium consisting of FeNH_4_(SO_4_) (100 μM), sulfuric acid (25 mM), xylenol orange (250 μM), and sorbitol (100 mM) and maintained in the dark for 30 min. Subsequently, sample absorbances were determined at 560 nm, and the values were then discounted from reactions performed in the absence of the plant extracts [75]. The H_2_O_2_ contents were then estimated based on a standard H_2_O_2_ curve and expressed as µmol H_2_O_2_ g^−1^ of fresh mass FM.

#### 4.2.6. Pigments, Flavonols, Nitrogen Balance Index, and Leaf Nutrient Contents

Chlorophyll (Chl, µg cm^−^²) and epidermal flavanol (Flv) contents and the anthocyanin index (Anth) were determined in leaves in the middle third of each plant using a leaf clip meter (Dualex^®^, Force-A, Orsay, France). The nitrogen balance index (NBI) was calculated according to the relation between Chl and Flv [76].

Calcium (Ca), magnesium (Mg), and potassium (K) contents were determined in *L. sativa* leaves collected at the end of the experimental period (40 days after KF application), which were then washed in distilled water and dried at 60 °C in a forced air circulation oven until reaching a constant mass. The dry material was then crushed in a Willey mill using a 20-mesh sieve, and elements were extracted by nitroperchloric digestion and quantified in accordance with Embrapa [77]. The Ca and Mg contents were determined using atomic absorption spectrophotometry, and the K contents were determined using flame photometry. All results are expressed as g kg^−1^ DM.

#### 4.2.7. Morphoanatomical and Histochemical Seed Characterization

Morphoanatomical and histochemical analyses were performed in seeds soaked in KF solutions at 0 mg L^−1^, 10 mg L^−1^, 20 mg L^−1^ and 30 mg L^−1^ for 48 h. For the morphoanatomical analysis, samples collected from the endosperm region were stored in a fixative solution as established by Karnovsky [78] for 24 h, and the material was prepared as described by Rodrigues et al. [69]. The samples were cross-sectioned (6 μm thickness) using a rotating microtome (1508R, Logen scientific, China) and stained with blue toluidine solution (0.05% 0.1 M phosphate buffer, pH 6.8) [79]. The histochemical detection of phenolic compounds was carried out in the central region of the last fully expanded leaf. Leaf fragments were fixed in a ferrous sulfate formalin solution [80] and the slide inclusion and preparation processes were carried out according to the characterization methodology. All images were obtained using an Olympus microscope (BX61, Tokyo, Japan) with a coupled CCD digital camera (DP-72, Olympus) using the bright field option.

### 4.3. Statistical Analyses

The obtained data were subjected to previous homogeneity (Levene test) and normality (Shapiro–Wilk test) error analyses. A one-way ANOVA was applied, and the means were compared using the Dunnett test considering *p* < 0.05 (*) and *p* < 0.01 (**). Log-transformed and auto-scaled (mean-centered and divided by the standard deviation of each variable) data were also assessed using a principal component analysis (PCA). Univariate analyses were performed using the *Assistat* software version 7.7 Beta, and multivariate analyses were carried out using the MetaboAnalyst software 5.0 (https://www.metaboanalyst.ca (accessed on 5 March 2022)).

## 5. Conclusions

The seed germination, morphophysiology, and metabolism of *L. sativa* plants were altered following exposure to different concentrations of KF. The main damage occurred after 40 days of exposure to simulated KF rain and irrigation. The plants absorbed the KF and, although no anatomical changes were observed, photochemical traits (Y_II_, ETR, and qL), lipid peroxidation (MDA), and nitrogen balance index (NBI) alterations occurred. These variables were proven to be potential F action indicators, especially in plants that do not display visible symptoms, such as *L. sativa*. This demonstrates that the analysis of this pollutant in the irrigation water used by farmers mainly for vegetable cultivation is paramount for the production of pollutant-free, non-toxic food items, thus ensuring greater food safety.

## Figures and Tables

**Table 1:** Total germination (TG) percentage, germination rate index (GRI), and root length (RL) of *Lactuca sativa* L. seedlings after 7 days of exposure to potassium fluoride (KF) at 0 mg L^−1^, 10 mg L^−1^, 20 mg L^−1^, and 30 mg L^−1^.

**KF (mg L^−1^)**	**TG (%)**	**GRI**	**RL (cm)**
0	93.00 ± 1.29	23.25 ± 0.32	3.31 ± 0.34
10	91.50 ± 2.22	22.88 ± 0.55	3.23 ± 0.27
20	86.00 ** ± 1.83	21.50 ** ± 0.46	2.91 ± 0.31
30	84.50 ** ± 0.96	21.13 ** ± 0.24	2.59 * ± 0.15
**One-Way ANOVA**			
**F (*t*-test)**	6.3077 **	6.3077 **	3.5409 *
***p***	**0.0081**	**0.0081**	**0.0185**

**Data represent the means ± SE (n = 4).** Asterisks indicate significant differences at 5% (*) and 1% (**) probabilities between the KF treatments and the control group according to the Dunnett test.

**Table 2:** Maximum photosystem II quantum yield (PSII) (F_v_/F_m_), effective PSII quantum yield (Y_II_), electron transport rate (ETR), photochemical extinction coefficient (q_L_), quenching of regulated non-photochemical dissipation (Y_NPQ_), photosynthetic rate (*A*), stomatal conductance (*g*s), transpiratory rate (*E*), relationship between internal and external CO_2_ concentrations (*C_i_/C_a_*), and carboxylation efficiency (*A/C_i_*) in *Lactuca sativa* L. plants after 40 days of exposure to 0 mg L^−1^, 10 mg L^−1^, 20 mg L^−1^, and 30 mg L^−1^ potassium fluoride (KF).

	**Chlorophyll *a* Fluorescence**				
**KF (mg L^−1^)**	**F_v_/F_m_**	**Y_II_**	**ETR**	**qL**	**Y_NPQ_**
0	0.83 ± 0.015	0.19 ± 0.008	101.5 ± 4.39	0.19 ± 0.03	0.60 ± 0.01
10	0.83 ± 0.003	0.13 ** ± 0.005	69.2 ** ± 2.80	0.13 ± 0.03	0.65 ± 0.03
20	0.81 ± 0.016	0.11 ** ± 0.017	57.7 ** ± 9.42	0.12 ± 0.05	0.65 ± 0.03
30	0.82 ± 0.009	0.13 ** ± 0.013	70.5 ** ± 7.24	0.12 ± 0.03	0.62 ± 0.03
**One-Way ANOVA**					
**F (*t*-test)**	**0.6919 ^NS^**	8.3466 **	8.3466 **	**1.0504 ^NS^**	**1.1407 ^NS^**
***P***	**0.5744**	**0.0028**	**0.0028**	**0.4059**	**0.3721**
	**Gas exchange**				
**KF (mg L^−1^)**	***A***	***g*_S_**	***E***	***C_i_/C_a_***	***A/Ci***
0	17.45 ± 3.07	0.68 ± 0.17	9.48 ± 2.05	0.83 ± 0.03	0.0524 ± 0.009
10	14.57 ± 1.45	0.67 ± 0.09	9.54 ± 1.03	0.87 ± 0.02	0.0410 ± 0.005
20	13.60 ± 3.00	0.68 ± 0.20	9.68 ± 2.18	0.87 ± 0.02	0.0407 ± 0.009
30	14.88 ± 1.66	0.63 ± 0.11	9.59 ± 1.44	0.86 ± 0.01	0.0450 ± 0.005
**One-Way ANOVA**					
**F (*t*-test)**	**0.4643 ^NS^**	**0.0268 ^NS^**	**0.0023 ^NS^**	**0.7642 ^NS^**	**0.6260 ^NS^**
***p***	**0.7125**	**0.9938**	**0.9998**	**0.5356**	**0.6118**

**Data represent the means ± SE (n = 4).** Asterisks indicate significant differences at 1% (**) probabilities between the KF treatments and the control group according to the Dunnett test. ^NS^, non-significant.

**Table 3:** Chlorophyll content (Chl), epidermal flavanol content (Flv), anthocyanin index (Anth), malonaldehyde (MDA), and hydrogen peroxide (H_2_O_2_) in *Lactuca sativa* L. leaves after 40 days of exposure to 0 mg L^−1^, 10 mg L^−1^, 20 mg L^−1^, and 30 mg L^−1^ potassium fluoride (KF).

KF (mg L^−1^)	Chl (µg/cm²)	Flv	Anth	MDA (nmol g^−1^ FM)	H_2_O_2_ (nmol g^−1^ FM)
0	14.58 ± 1.16	0.33 ± 0.01	0.60 ± 0.02	4.03 ± 0.27	5.46 ± 0.83
10	11.82 ± 0.50	0.32 ± 0.03	0.59 ± 0.01	5.82 * ± 0.26	5.32 ± 0.71
20	10.77 * ± 0.19	0.31 ± 0.01	0.60 ± 0.02	6.04 * ± 0.33	5.49 ± 0.42
30	11.50 * ± 0.86	0.31 ± 0.04	0.60 ± 0.01	6.26 * ± 0.68	6.97 ± 0.56
**One-Way ANOVA**					
**F (*t*-test)**	4.6145 *	**0.1135 ^NS^**	**0.0884 ^NS^**	5.9450 *	**1.4373 ^NS^**
***p***	**0.013**	**0.9512**	**0.9656**	**0.01**	**0.2806**

**Data represent the means ± SE (n = 4).** Asterisks indicate significant differences at 5% (*) probabilities between the KF treatments and the control group according to the Dunnett test. ^NS^, non-significant.

**Table 4:** Nitrogen balance index (NBI) and calcium (Ca), magnesium (Mg), and potassium (K) levels in *Lactuca sativa* L. leaves after 40 days of exposure to 0 mg L^−1^, 10 mg L^−1^, 20 mg L^−1^, and 30 mg L^−1^ potassium fluoride (KF).

KF (mg L^−1^)	NBI	Ca (g kg^−1^)	Mg (g Kg^−1^)	K (g Kg^−1^)
0	42.63 ± 2.27	14.93 ± 0.56	5.85 ± 0.57	52.00 ± 0.82
10	34.46 ** ± 0.83	15.25 ± 0.42	5.80 ± 0.08	51.83 ± 1.50
20	35.91 ** ± 1.78	14.10 ± 0.57	5.55 ± 0.23	51.80 ± 1.38
30	35.43 ** ± 3.33	14.63 ± 0.67	5.45 ± 0.17	51.98 ± 1.37
**One-Way ANOVA**				
**F (*t*-test)**	5.1728 **	**0.7517 ^NS^**	**0.3631 ^NS^**	**0.0062 ^NS^**
***p***	**0.0082**	**0.5422**	**0.7809**	**0.9993**

**Data represent the means ± SE (n = 4).** Asterisks indicate significant differences at 1% (**) probabilities between the KF treatments and the control group according to the Dunnett test. ^NS^, non-significant.

## Supplementary Materials

The following supporting information can be downloaded at: https://www.mdpi.com/article/10.3390/plants11233406/s1. Figure S1: Visual features of Lactuca sativa leaves after 40 days of exposure to simulated rain with potassium fluoride at different concentrations: (**A**) control, (**B**) 10 mg L^−1^ KF, (**C**) 20 mg L^−1^ KF and (**D**) 30 mg L^−1^ KF. Scale bar 7 cm. Figure S2: Accumulation of phenolic compounds marked in black color in the roots and leaves of Lactuca sativa after 40 days of exposure to simulated rain of potassium fluoride at different concentrations. (**A,B**) control, (**C,D**) 10 mg L^−1^ KF, (**E,F**) 20 mg L^−1^ KF and (**G,H**) 30 mg L^−1^ KF. (**A–G**) 200 µm scale bar. (**H**) 500 µm scale bar. Yellow arrows indicate accumulation of phenolic compounds.
