# Supplementary material for: Morphoanatomical, Physiological, and Biochemical Indicators in Lactuca sativa L. Germination and Growth in Response to Fluoride
Source: Plants (Basel). 2022 Dec 6;11(23):3406. doi: 10.3390/plants11233406 (PMC9738690; doi:10.3390/plants11233406)
Supplement: Supplementary file 1 [file plants-11-03406-s001.zip › plants-2012178-supplementary.pdf]

Supplementary Material

Morphoanatomical, physiological and biochemical indicators in *Lactuca sativa* L. germination and growth in response to fluoride

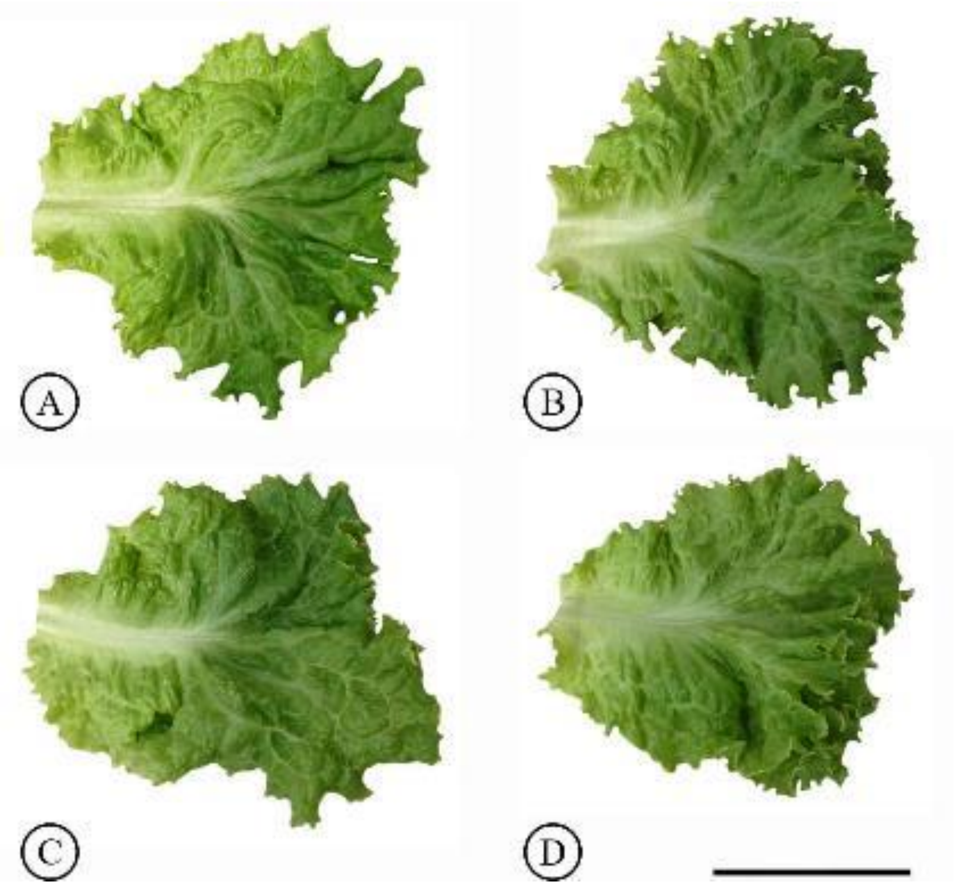

Figure S1. Visual features of *Lactuca sativa* leaves after 40 days of exposure to simulated rain with potassium fluoride at different concentrations: (A) control, (B) 10 mg L^{-1} KF, (C) 20 mg L^{-1} KF and (D) 30 mg L^{-1} KF. Scale bar 7 cm.

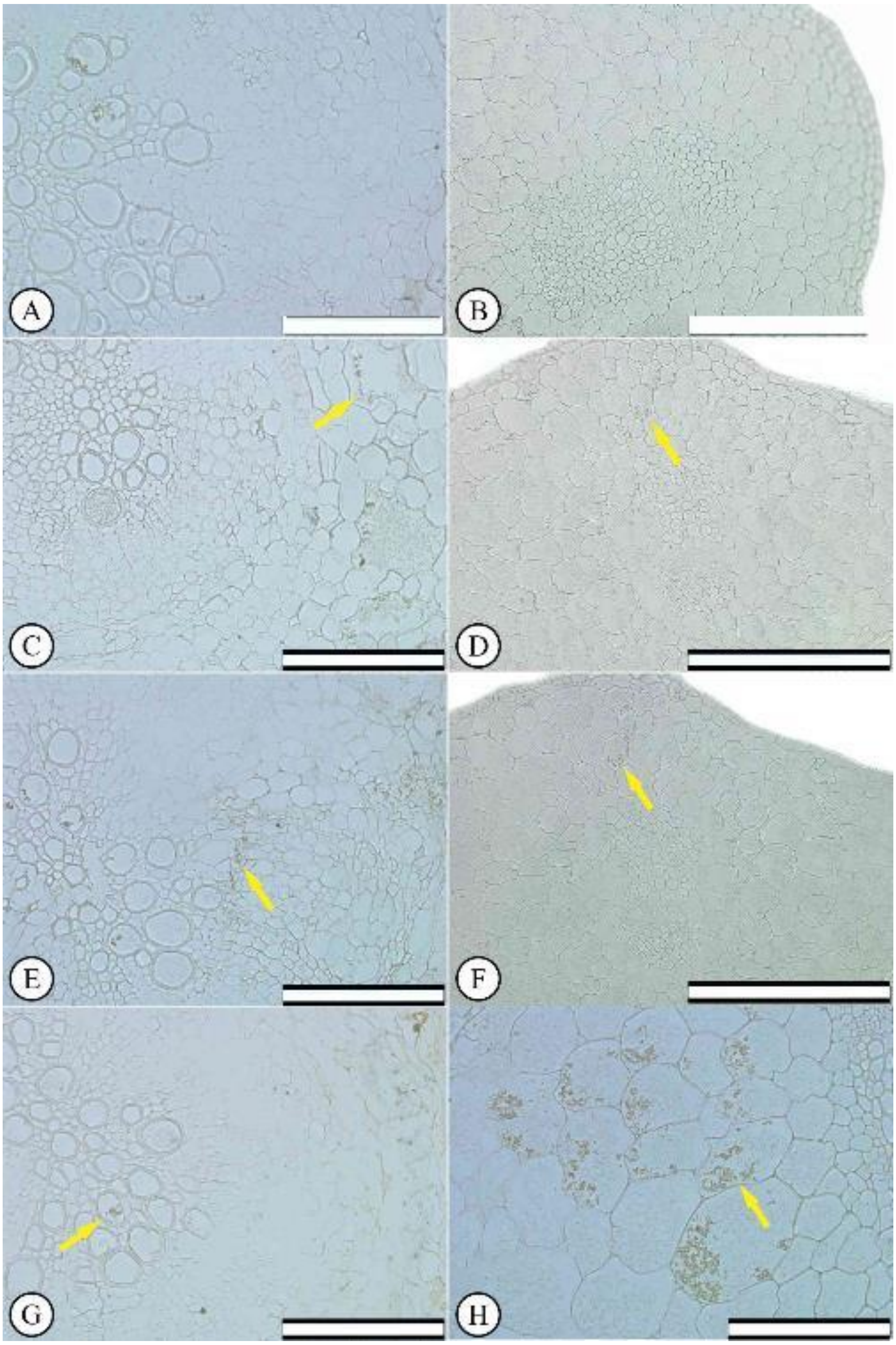

Figure S2. Accumulation of phenolic compounds marked in black color in the roots and leaves of *Lactuca sativa* after 40 days of exposure to simulated rain of potassium fluoride at different concentrations. (A-B) control, (C-D) 10 mg L^{-1} KF, (E-F) 20 mg L^{-1} KF and (G-H) 30 mg L^{-1} KF. (A-G) 200 µm scale bar. (H) 500 µm scale bar. Yellow arrows indicate accumulation of phenolic compounds.
